# Enhancing labour efficiency in coffee harvesting using process mining and statistical analysis

**DOI:** 10.1186/s12870-026-09212-3

**Published:** 2026-06-19

**Authors:** Sridevi Saralaya, Anjali Ganesh, Ravikantha Prabhu, Roopesh Kumar, Manoj Kumar, Shweta Gakhreja, Ruchi Singh Rajawat, Shefali Goel

**Affiliations:** 1 Department of Computer Science and Engineering, St Joseph Engineering College, Mangaluru, India; 2 Department of Business Administration, St Joseph Engineering College, Mangaluru, India; 3 Department of Mechanical Engineering, St Joseph Engineering College, Mangaluru, India; 4 Department of Mechanical Engineering, ABES Engineering College, Ghaziabad, 201009 India; 5 Manipal University Jaipur, Jaipur, India; 6 Department of Mathematics, Institute of Applied Sciences and Humanities, GLA University, Mathura, Uttar Pradesh 281406 India; 7 Department of General Management, IILM, Lodhi Road, New Delhi, India

**Keywords:** Coffee harvesting, Coffea Arabica, Arabica, Coffea Canephora, Robusta, Agricultural data analysis, Process mining, SPSS, Labour productivity, Worker efficiency

## Abstract

**Background:**

Coffee processing, in wet processing environments, where operational challenges are prevalent, is a pivotal aspect in product quality and market value. The harvesting and processing of coffee in Sakaleshpur (Arabica) and Chikkamagaluru (Robusta) are examined in this study. It focuses on 15 batches of Robusta harvesting and three different stages of Arabica harvesting: fly picking, mid harvesting, and stripping. The analysis of process features and productivity-impacting factors is conducted with the help of statistical analysis and Process Mining (PM).

**Findings:**

The results indicate that there is a strong negative correlation between the number of workers and the quantity of coffee picked in the process of fly picking, that is, the increased number of labour does not necessarily lead to increased output. Statistical analysis has shown that labour efficiency, weather and gender are key factors affecting the harvesting performance. Although cause-and-effect research can determine significant factors influencing operational efficiency, PM can identify variations in workflow patterns.

**Conclusions:**

The paper demonstrates that integrating PM and statistical techniques can provide informative data on the way coffee harvesting takes place. The results highlight how important it is to manage labour effectively and take human and environmental aspects into account in order to increase productivity. This method provides a data-driven paradigm for increasing coffee processing systems' efficiency.

## Introduction

Coffee production supports the livelihoods of approximately 125 million people, almost 80% of them being smallholder farmers, and a significant source of global farm-based economy [1]. The most common types of coffee grown in India which is one of the largest coffee exporting countries are coffea arabica and Coffea canephora (Robusta) [2, 3]. Arabica is preferred due to its better taste and higher price in the market. Nevertheless, harvesting and post-harvest processing are labour-intensive, seasonal, and environmental factors create several challenges to coffee production. Proper drying and storage are important in maintaining quality since coffee is harvested within a short period of time but is consumed throughout the year [4–6].

Most harvesting processes are performed manually and involve fly picking, mid-harvesting and stripping as well as methods such as selective and strip picking [7–10]. Selective selection ensures quality, but is more time-consuming and labour-intensive; the yield of workers is 45 to 55 kg per day on average [11, 12]. After harvesting the beans undergo several processing steps such as pulping, fermentation, washing and drying, which must be well co-ordinated to ensure quality is maintained [13–16]. Although it is important, workflow analysis is often not done in a thorough manner leading to inefficiencies and inconsistency in the processes.

Recent developments in digital and analytical technologies have significantly improved agricultural practices through data-driven decision-making and operational efficiency. AgriTech solutions such as Geographic Information Systems, drones, and remote sensing have been widely applied in agriculture [17–20]. In the coffee sector, several digital solutions have been developed for improving administration, traceability, and supply chain monitoring, including web-based management systems, blockchain-based transparency platforms, and agile-based monitoring systems [21–23]. They are web-based sales and operations management systems, blockchain based transparency systems, agile-based software supply chain monitoring systems [21, 24, 25]. However, most existing studies primarily focus on traceability, monitoring, and management applications, with limited attention to detailed workflow analysis and labour productivity assessment. In this context, the present study introduces a novel integration of Process Mining (PM) with statistical and cause effect analysis to model coffee harvesting workflows, identify bottlenecks, and analyse factors affecting labour efficiency in a more systematic and data-driven manner.

PM has become a helpful tool of analysis in data science and business process management. It allows event logs to be used in locating and analysing real-world processes, aiding in determining inefficiencies and deviations of workflows on the expected processes [26]. This technique has found widespread application in manufacturing and education [27–29], in healthcare to explore treatment courses and adherence [10, 30–33]. It is yet to be utilized in agricultural practices though and particularly in harvesting and processing coffee.

Previous studies on coffee production have not given much focus to workflow-level analysis, largely focusing on post-harvest strategies and agronomic strategies. There is a lack of research that utilizes PM to evaluate harvesting activities of both Arabica and Robusta species. Moreover, the integrative use of PM and statistics to determine labour efficiency and the influence of different operational and environmental factors has not been researched enough. In particular, PM has not been extensively applied in modelling, analysing and optimising coffee harvesting, and combining with statistical methods to assess labour efficiency and influencing factors has not been adequately studied. To seal this gap, the present research employs PM as one of the basic tools of analysis to examine coffee harvesting practices in the Karnatka, India, districts of Sakaleshpur and Chikkamagaluru.

To develop overall process models that would reflect activity chains, durations, and variations due to all controllable (such as labour management and mechanisation) and uncontrollable (such as environmental conditions) factors, this research integrates statistical analysis with PM tools such as Disco and ProM. It is interesting to note that this work combines PM and statistical analysis and cause-effect analysis to give an in-depth, data-driven view of coffee harvesting activities, unlike earlier agricultural research projects, which typically relied on the conventional observational or statistical analysis. This combined method is more comprehensive and analytical than traditional methods because it allows mapping both real process flows and systematically identifying inefficiencies, bottlenecks and determining key productivity factors.

The objectives of this study are as follows:

1. To analyse the wet processing workflow of Arabica coffee with emphasis on the fly-picking stage.
2. To examine the harvesting and processing practices of Robusta coffee.
3. To evaluate the relationship between labour input and coffee yield.
4. To assess the impact of labour, environmental, and other factors on variations in coffee picking.

In Objectives 1 and 2, workflow analysis was carried out using PM techniques to systematically examine operational sequences and identify process inefficiencies in coffee harvesting activities. Unlike previous studies that mainly focused on isolated productivity indicators or conventional observational approaches, the present work integrates PM-based workflow modelling with statistical labour efficiency analysis to provide a more comprehensive evaluation framework. In Objectives 3 and 4, labour efficiency and the factors affecting harvesting performance were quantitatively evaluated using statistical tools. The study is theoretically grounded in labour productivity and efficiency analysis concepts, where workforce output is influenced by operational, environmental, and human-related variables. In particular, the principle of diminishing marginal productivity provides the conceptual basis for examining variations in labour contribution under different workforce and field conditions. Furthermore, the major factors influencing harvesting performance were systematically identified and analysed using a fishbone diagram, enabling the integration of empirical findings with root-cause analysis. This combined methodological framework represents the novelty of the study by linking process-oriented workflow analysis, statistical efficiency evaluation, and causal factor identification within the specific application domain of coffee harvesting operations.

## Methodology

**Fig. 1:**
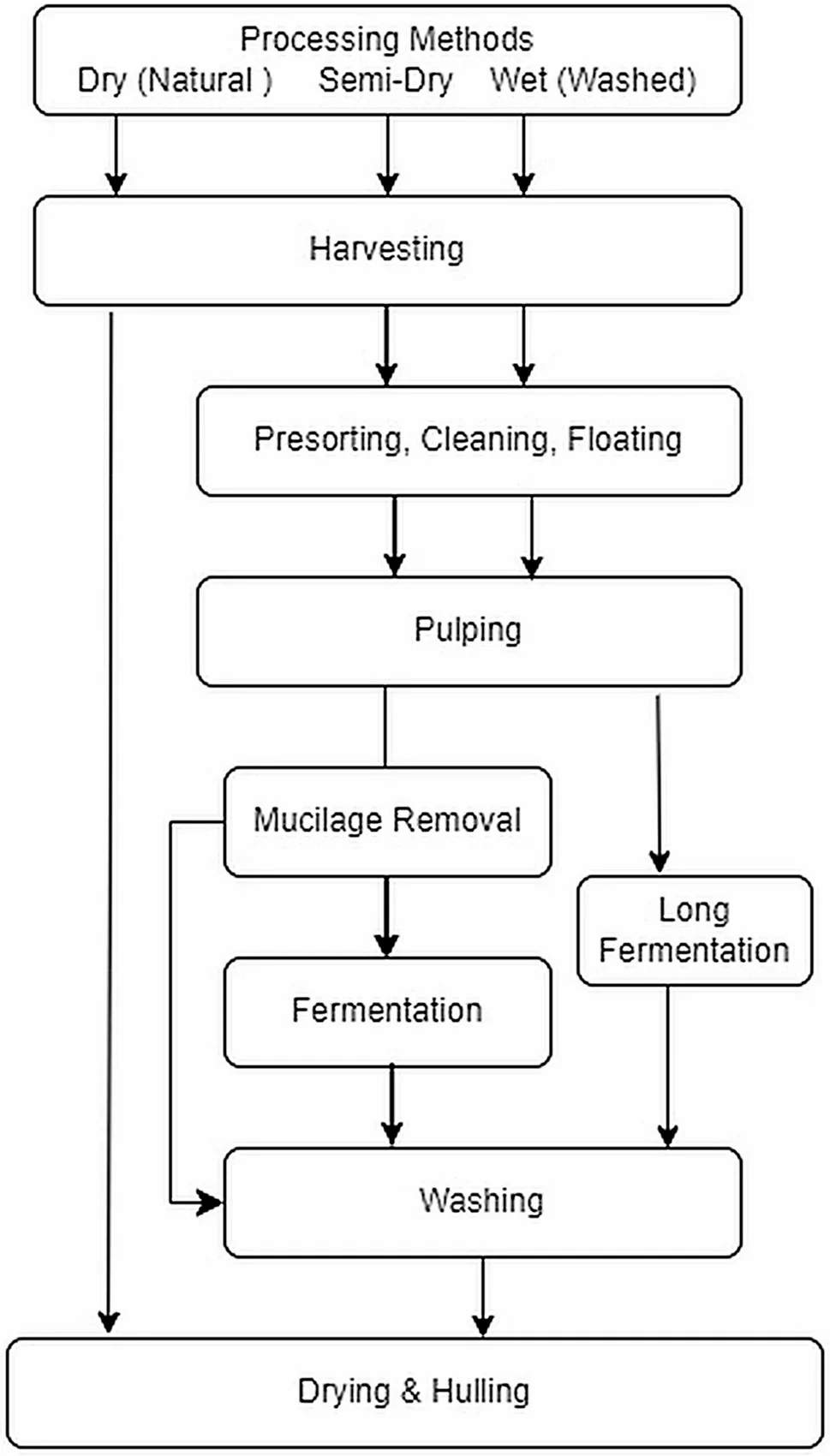
Coffee process method [34]

Coffee processing involves a sequence of interrelated activities that directly influence the quality and efficiency of the final output. Understanding these operational steps is essential for identifying delays, bottlenecks, and process deviations. In this context, the steps involved in coffee processing are illustrated in Fig. 1 [34].

In this study, PM techniques were applied to analyse the timing and control flow of activities involved in coffee harvesting. When the start and end times of each activity are recorded, key PM techniques such as discovery and conformance checking can be employed. The discovery technique reconstructs event data into a visual representation of workflows performed by organisational resources [35]. Several PM tools are available, including Apromore, ARIS, BusinessOptix, ProM, Disco, Celonis, Everflow, Lana, and Minit [36].

In this study, Disco and ProM were selected to discover process models and analyse control flow and activity sequences [37, 38]. These tools were chosen for their complementary strengths in visualization, performance analysis, and advanced analytical capabilities such as plugin support and conformance checking. Event logs were preprocessed by identifying case IDs, sorting events chronologically, and removing incomplete or duplicate entries. The logs included essential attributes such as Case ID (BatchNo), Event (activity), and Timestamp (start and end time), along with EventId for data integrity. Activities recorded included sorting, weighing, pulping, fermentation, washing, drying, winnowing, moisture checking, packing, and dispatch.

### Data collection and sampling strategy

Data were collected from coffee plantations in Sakaleshpur and Chikkamagaluru, Karnataka, India, using a purposive sampling strategy. These regions were selected due to their prominence in Arabica and Robusta coffee production and their representation of typical harvesting practices in South India. Within each region, estates were selected based on accessibility, willingness to participate, and operational scale to ensure inclusion of diverse harvesting conditions. For statistical analysis, the unit of analysis was defined at the individual worker level. The 504 worker-based observations were collected from 15 batches of flies used in Arabica coffee picking. For all the batches collected, worker attendance and plucking per individual was noted on a daily basis by the supervisors at the estate. Any worker involved in a particular batch was counted as an observation and his/her individual performance (kg plucked), gender, and weather condition on that particular day were documented. In total, there were 504 observations, meaning that all workers participating in any batch were documented as an observation each. There was a purposeful breakdown from aggregate data on batch level to individual worker data on each working day. This made statistical inference easier from the individual perspective rather than from batch levels. Transition to individual level of analysis from aggregate was explicitly stated during data preparation phase.

As mentioned earlier, purposive sampling technique was employed for the purpose of conducting the present study since the objectives of the study entailed collecting data from plantations practicing both Arabica and Robusta coffee harvests. This was an alternative to random sampling owing to the fact that the study had limited options in terms of accessing all kinds of information from all the estates since not all of them kept systematic records at a batch level, nor did all of them practice all kinds of harvests. This meant that purposively selected estates needed to be those who practiced both Arabica and Robusta coffee harvests in order to meet the objective of conducting the study.

To minimise potential bias arising from reliance on plantation records and interviews, data were collected through multiple sources, including plantation logs, direct field observations, and interactions with supervisors and workers. The recorded activity timings were cross-verified in situ wherever feasible, and inconsistencies were resolved through repeated clarification with estate personnel. In addition, only those batches with complete and consistent time records across all activities were included in the final dataset, thereby improving data reliability.

Sampling was conducted at three levels: (i) estates, (ii) harvesting batches, and (iii) individual workers. A total of 34 batches were analysed, comprising 19 Arabica batches (15 fly-picking, 2 mid-harvesting, and 2 stripping) and 15 Robusta batches. These batches were intentionally chosen to capture variability across harvesting stages and crop types. A snapshot of activities performed in one batch of fly picking is displayed in Table 1.

**Table 1:** Activities performed in one batch of fly picking

Event ID	Batch Nos	Activity	Start date and time	End date and time
1	C1B1	Fly Picking	27-10-2024, 08:30	27-10-2024, 16:30
2	C1B1	Sorting	27-10-2024, 16:30	27-10-2024, 16:50
3	C1B1	Weighing	27-10-2024, 16:50	27-10-2024, 17:00
4	C1B1	Separation of Single bean & dried coffee	27-10-2024, 17:00	27-10-2024, 18:00
5	C1B1	Pulping	27-10-2024, 18:00	27-10-2024, 22:00
6	C1B1	Natural Fermentation	27-10-2024, 22:00	28-10-2024, 06:00
7	C1B1	Washing	28-10-2024, 06:00	28-10-2024, 08:00
8	C1B1	Drying	28-10-2024, 08:00	04–11-2024, 16:30
9	C1B1	Winnowing	04–11-2024, 16:30	04–11-2024, 17:00
10	C1B1	Moisture checking	04–11-2024 17:00	04–11-2024, 17:10
11	C1B1	Packing and Dispatch	04–11-2024, 17:10	04–11-2024, 18:30

To further ensure consistency, timestamps were standardised into a uniform format, and chronological ordering of events was verified during pre-processing. Any missing or overlapping time entries were carefully screened and excluded to avoid distortion in PM results.

### Event log preparation and PM

**Fig. 2:**
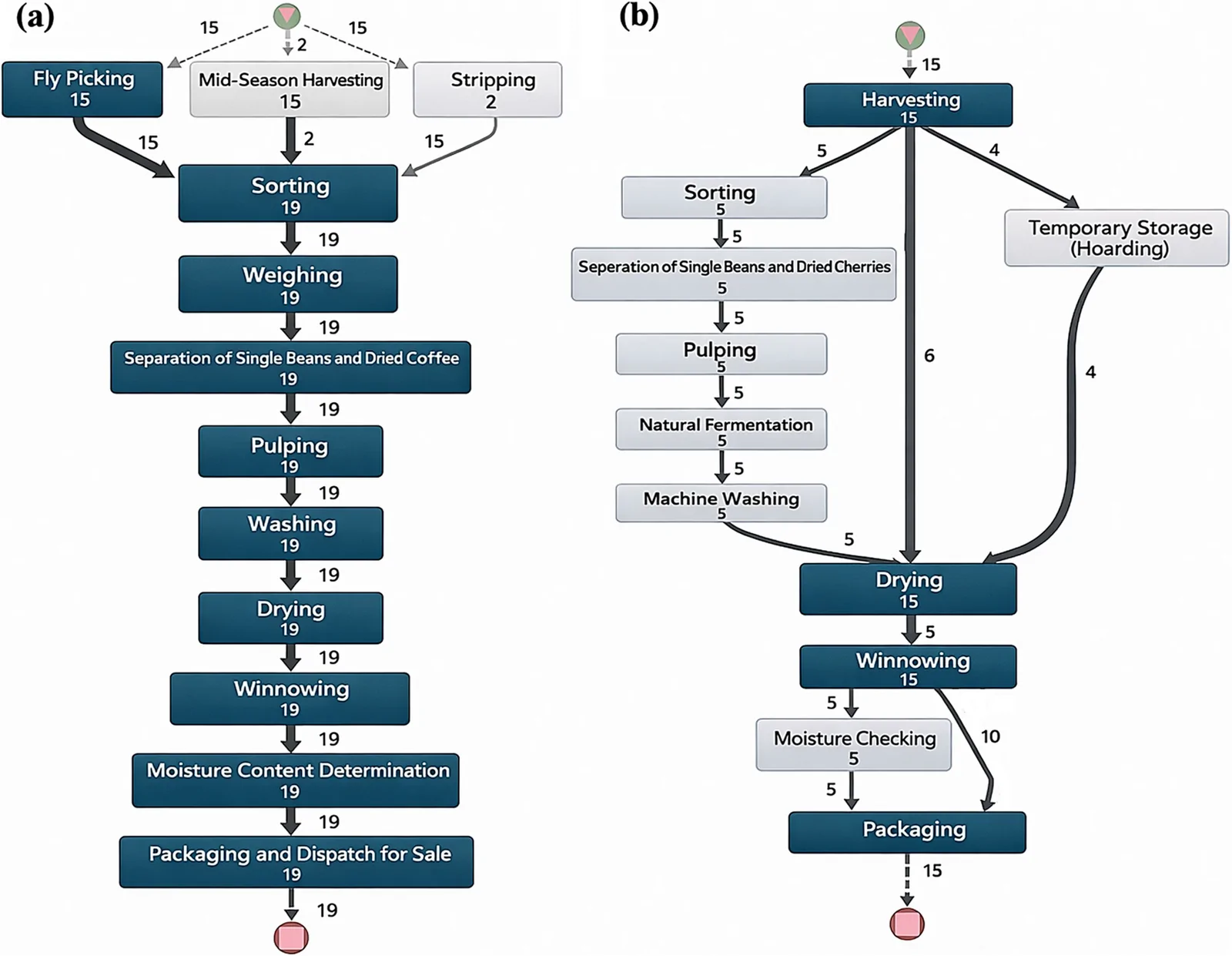
Process models of coffee harvesting generated using the Disco tool: (**a**) Arabica, (**b**) Robusta

The coffee harvesting event log was developed by recording the start and end times of each activity across batches. Standard PM pre-processing steps, including case identification, timestamp alignment, and data cleaning, were applied. Variant filtering with a noise threshold of 20% and default discovery algorithms in Disco and ProM were used to generate process models and analyse activity sequences and durations. The process maps generated for 19 Arabica batches and 15 Robusta batches (Fig. 2) reveal distinct workflow patterns. The Arabica workflow (Fig. 2(a)) exhibits a strictly sequential structure, where all activities are mandatory. The process includes 15 fly-picking batches and 2 batches each of mid-harvesting and stripping, indicating a consistent execution pattern. In contrast, the Robusta workflow (Fig. 2(b)) shows higher variability. Among the 15 batches, 5 batches follow the wet processing method similar to Arabica, while 10 batches adopt the dry processing method, skipping the pulping stage. Additionally, 4 batches involved hoarding for durations ranging from 1 to 4 days between November 29, 2024, and December 2, 2024.

### Identification of causative factors

Causative factors influencing coffee harvesting performance were identified through a combination of literature review, field observations, and stakeholder interactions. These factors were systematically organised using an Ishikawa (fishbone) diagram, which facilitated structured categorisation.

The factors were grouped into four major categories:

(i) environmental constraints,
(ii) people-related factors,
(iii) uncontrollable variables, and.
(iv) lack of mechanisation/technology.

This classification enabled a comprehensive and structured analysis of the determinants affecting labour productivity.

### Statistical analysis and justification

To complement the PM data, statistical analysis was performed with the help of IBM SPSS Statistics, and each of the techniques was selected based on the objectives of the study. The primary aim of using PM was to analyze the wet processing process of Arabica coffee and the fly-picking step in particular and to examine the approaches to Robusta harvesting and processing applying the profound workflow discovery and change analyses. The efficiency of the labour was evaluated statistically and the factors influencing Objectives 3 and 4 were identified. To determine the strength and direction of the relationship between labour input and coffee yield (Objective 3), Pearson correlation analysis was employed. This method was selected because both variables are continuous in nature and preliminary data screening indicated approximate linearity and normal distribution, satisfying the assumptions required for Pearson’s correlation analysis. To analytically evaluate the differences in coffee-picking productivity among labour groups and working environments (Objective 4), independent sample t-tests and one-way analysis of variance (ANOVA) were employed based on the nature of the variables and group comparisons involved. The t-test was used when comparing the mean productivity between two labour categories, while one-way ANOVA was applied to assess productivity variations across multiple labour groups and different working environments. These statistical techniques were selected because they enable the identification of statistically significant differences in mean productivity levels and provide a reliable basis for understanding the influence of labour characteristics and workplace conditions on coffee-picking performance. The chi-square test was used to examine the relationship between categorical variables, including gender and task allocation, which provided information about the trends of labour allocation that influence the harvesting performance. The relationship and impact aspects outlined under Objectives 3 and 4 were examined using a multiple linear regression model. In this analysis, total coffee-picking output was considered the dependent variable, as it represents the productivity outcome being predicted, whereas workforce size, gender, and environmental conditions were treated as independent variables because they are the influencing factors expected to affect productivity. The model was employed to evaluate the individual and combined effects of these variables on overall output and to identify the factors that significantly contribute to productivity variations. Additionally, a fishbone diagram was employed to methodically determine and examine the underlying factors affecting harvesting efficiency.

The number of workers variable in the correlation and regression tests is the number of workers in a certain batch in a harvesting day that was taken at the batch level and then integrated with individual records. As such, because each worker’s performance was captured individually and each worked in a different section of the farm, individual cases can be considered as independent statistical events. It is noteworthy that the study design involved treating each individual record of a worker-day as an observation unit with no outcome measure overlapping between workers in the same batch. In addition, workers who were observed in multiple batches were also regarded as individual observations since their environments (weather, field section, other workers) were different from those in other batches. Indeed, Levene’s tests showed homogeneity of variance where necessary, and VIFs did not exceed 5 in any regression models.

Disco and ProM were used to examine event logs for PM in order to find workflow models and pinpoint process variances. Disco employed filtering thresholds to retain the most prevalent pathways and activities which increases clarity of a model without losing important behavioural patterns. ProM employed standard process discovery algorithms with parameter selections that provide a trade-off between model complexity and interpretability. To help make sure that the models reflect observed processes without overgeneralisation, model validation was conducted by evaluating the agreement between event logs and identified models, as well as fitness and precision. These techniques together provide a powerful analysis package whereby statistically validated assessment is coupled with workflow discovery to comprehensively review labour efficiency and process performance.

### Assumptions and validation

The assumptions underlying the statistical analyses were systematically evaluated prior to hypothesis testing. Independence of observations was ensured through worker-level data structuring.

Normality of the data distribution was assessed using both descriptive statistics (skewness and kurtosis) and formal tests such as the Shapiro–Wilk test. Homogeneity of variance was examined using Levene’s test for t-tests and ANOVA. Linearity and homoscedasticity assumptions for regression were evaluated using residual plots, and multicollinearity was checked using variance inflation factors (VIF).

These diagnostic checks confirmed that the data satisfied the assumptions required for parametric testing. Where minor deviations were observed, the robustness of the selected tests ensured the validity of the results.

Data reliability was strengthened through cross-verification of plantation records, field observations, and stakeholder inputs. Although potential limitations such as manual recording errors or recall bias may exist, these were minimised through triangulation of multiple data sources and consistent validation procedures.

## Results

**Fig. 3:**
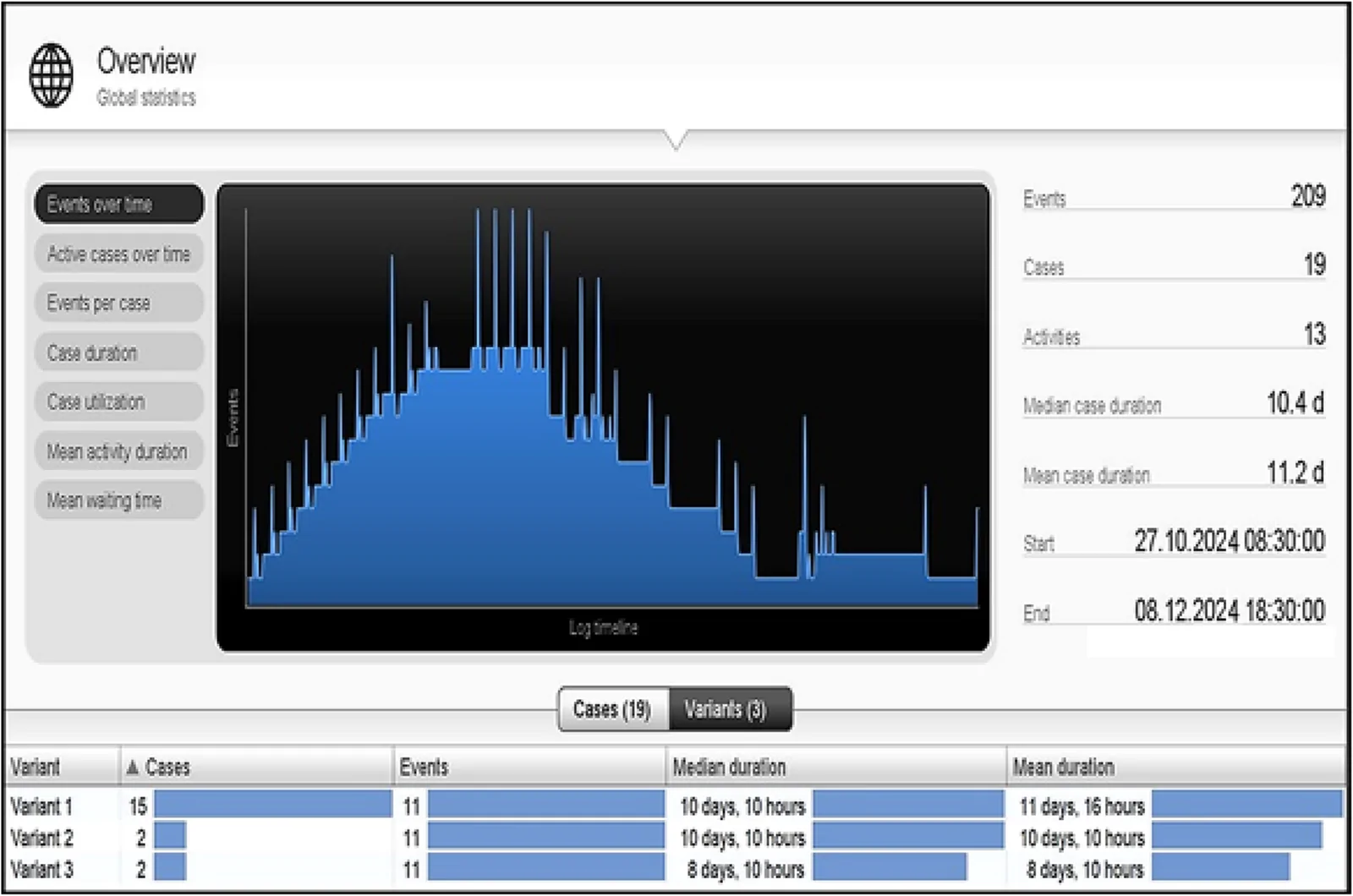
The three variants of arabica coffee plucking

In this section, the results obtained using the PM Disco tool and SPSS are discussed. Figure 3 presents an overview of the event log, comprising 209 events across 19 cases and 13 activities, with a mean case duration of 11.2 days. The analysis identifies three process variants of Arabica coffee harvesting, corresponding to fly picking, mid-harvesting, and stripping. Among these, Variant 1 is the most frequent, accounting for the majority of cases, while Variants 2 and 3 occur less frequently with comparatively shorter mean durations. These variants reflect differences in harvesting practices and workflow execution within the dataset.

**Fig. 4:**
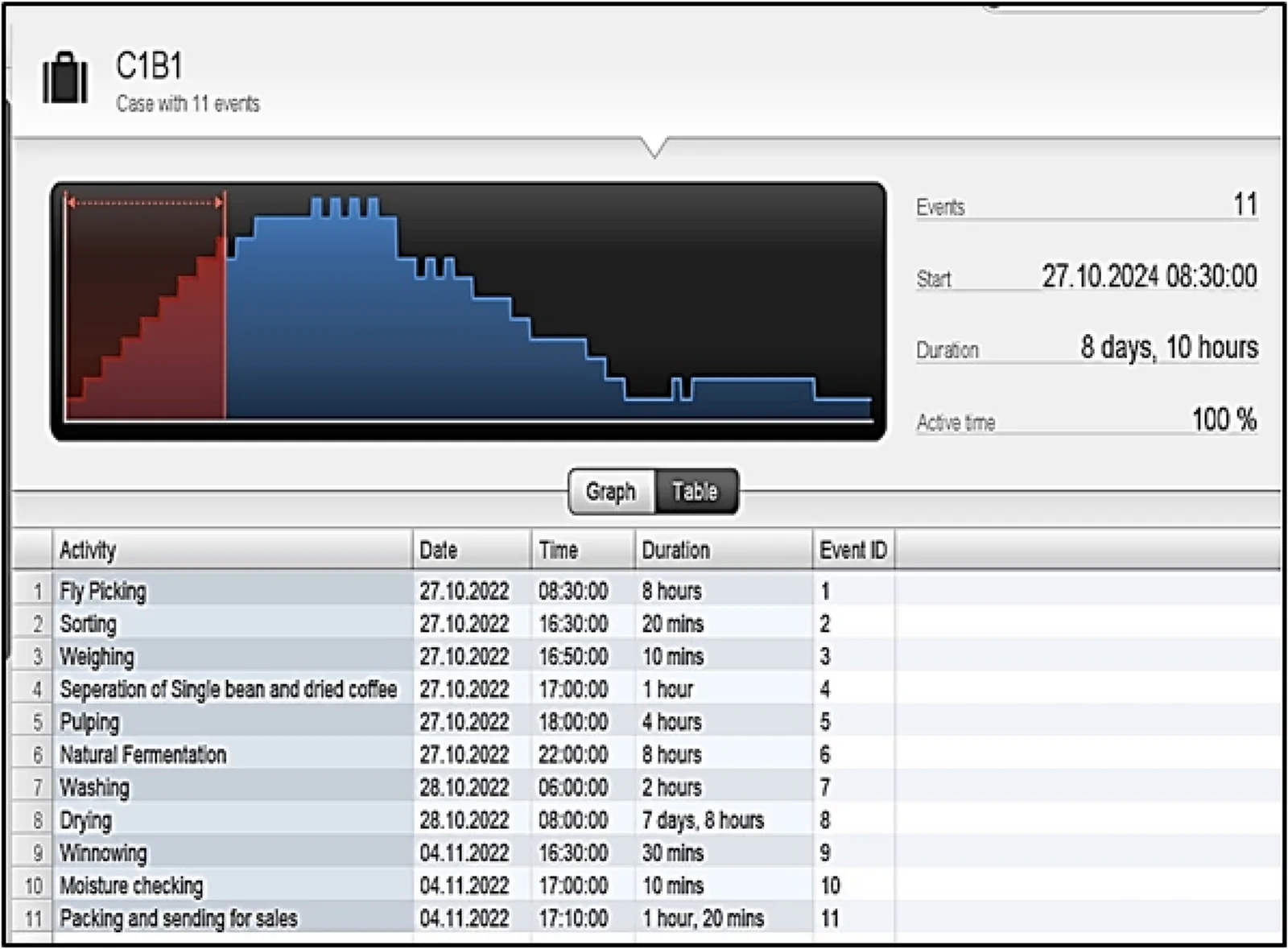
Sequence of activities during harvesting of Arabica

Figure 4 presents the event log of a representative case (C1B1), illustrating the sequence of activities, timestamps, and corresponding durations in the Arabica coffee harvesting process. The results show that a total of 11 events were recorded, with the process commencing on 27.10.2024 at 08:30 and spanning 8 days and 10 h, with 100% active time indicating a continuous workflow without significant idle periods. The activity sequence progresses from fly picking through sorting, weighing, separation of single beans and dried coffee, pulping, and natural fermentation, followed by washing and drying, where drying is identified as the most time-intensive stage, accounting for approximately 7 days and 8 h of the total duration. The process concludes with winnowing, moisture checking, and packing for dispatch. While the sequence of activities remains consistent across variants, the results indicate variations in the duration of key stages, particularly drying and fermentation, which significantly influence the overall process time, whereas stripping exhibits shorter durations and fly picking and mid-harvesting require comparatively longer processing times.

**Fig. 5:**
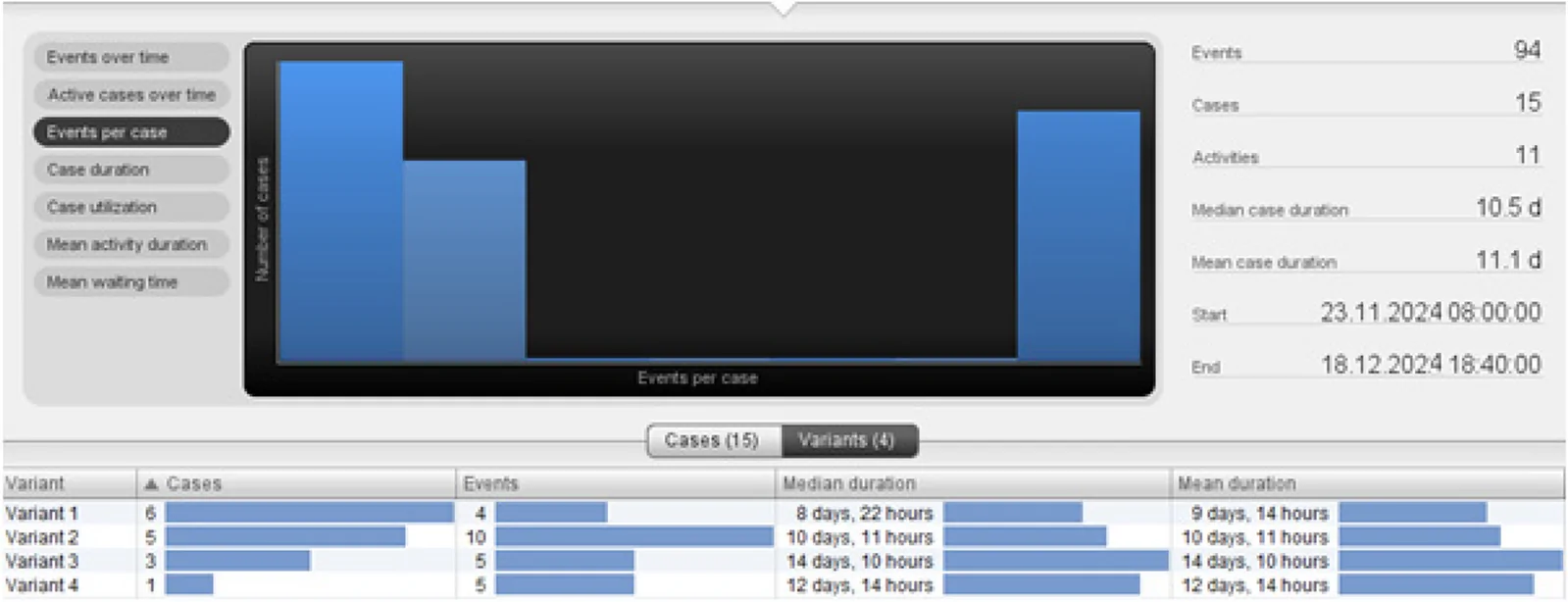
The four variants of coffee plucking - Robusta

**Fig. 6:**
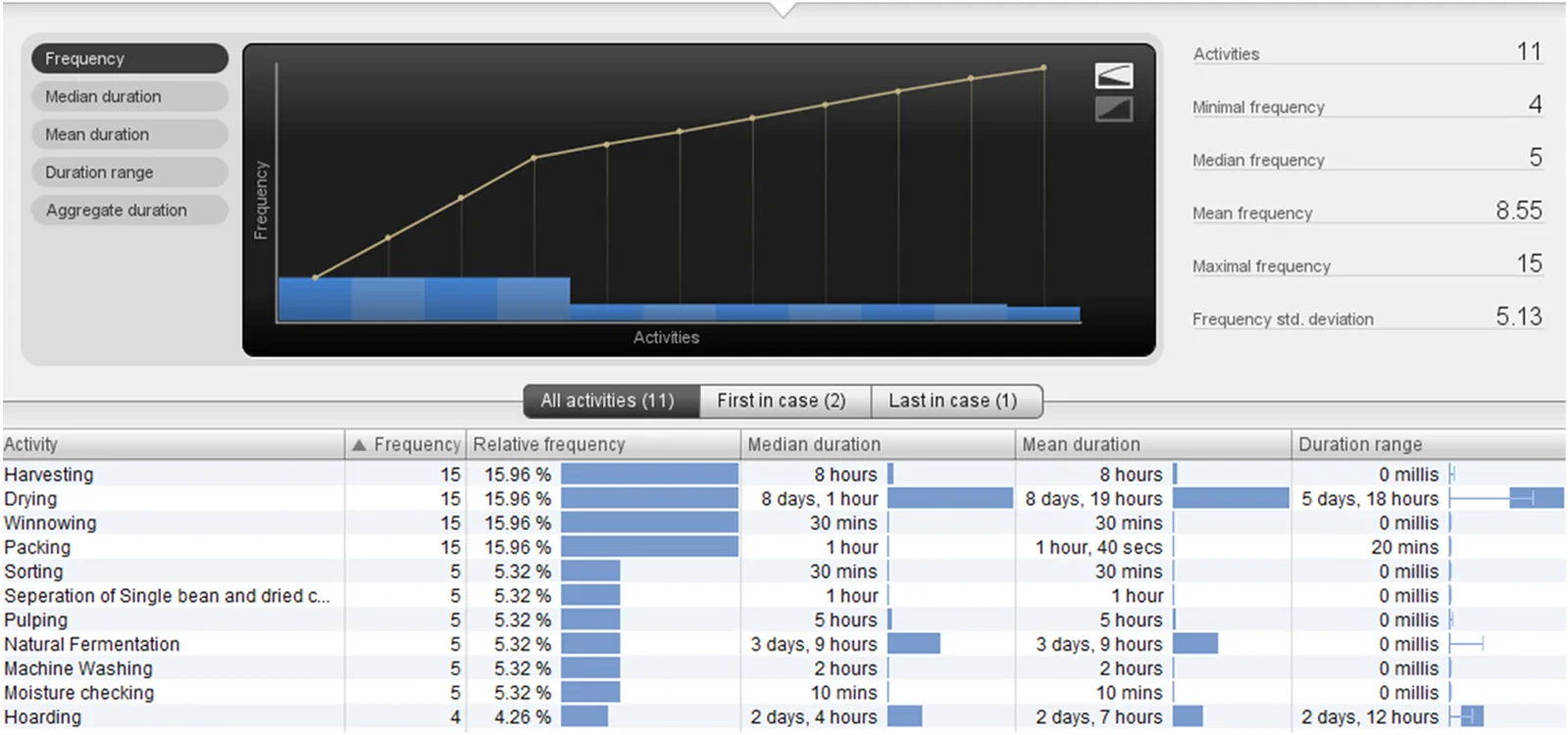
Activities in one batch of Robusta (Variant 1)

Figure 5 illustrates four variants of Robusta harvesting. Variant 1 represents six cases with four events (Harvesting, Drying, Winnowing, and Packing), with a mean duration of 9 days and 14 h. Variant 2 consists of five cases with ten events, representing the wet method of processing Robusta, as shown in Fig. 6. Variants 3 and 4 follow the same sequence of five events (Harvesting, Hoarding, Drying, Winnowing, and Packing) but exhibit variation in duration. Variant 4 differs primarily in drying time, with a shorter duration of 12 days and 14 h compared to approximately 14 days and 10 h for the other variants. Figure 6 presents the sequence of activities in one batch of Robusta harvesting, including the frequency and mean duration of each activity. Although the relative frequencies of activities such as harvesting, drying, winnowing, and packing remain similar across variants, drying shows the highest mean duration. Under normal estate conditions, the drying process typically takes about 8 to 9 days.

**Fig. 7:**
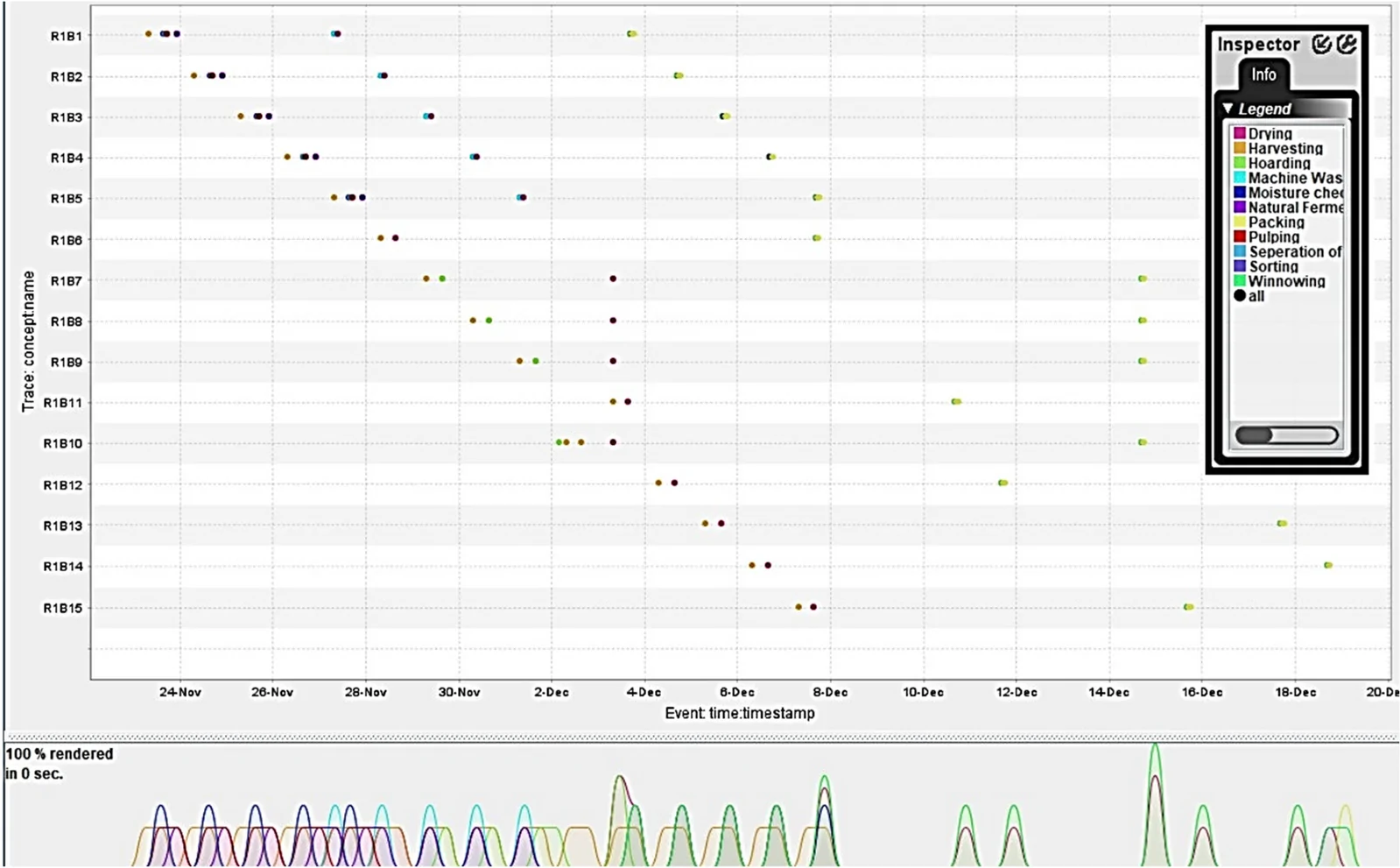
Batch sequence during the harvesting period for 15 Robusta batches

The Dotted Chart feature within the ProM PM tool was used to analyse temporal work patterns by visualising activity timelines and scheduling behaviour (Fig. 7). The results provide a detailed representation of the sequence and duration of activities across multiple batches, where each dot corresponds to a specific activity performed on a given day. This enables clear identification of process durations and overlaps. For instance, the first five batches (R1B1 to R5B5) each exhibit ten events, corresponding to Variant 2, whereas batch R1B10 follows Variant 4, with its workflow spanning from 2 December 2024 to 14 December 2024 at approximately 18:30 h. Overall, the dotted chart facilitates comparison of workflow patterns and highlights variations in process execution across batches.

**Fig. 8:**
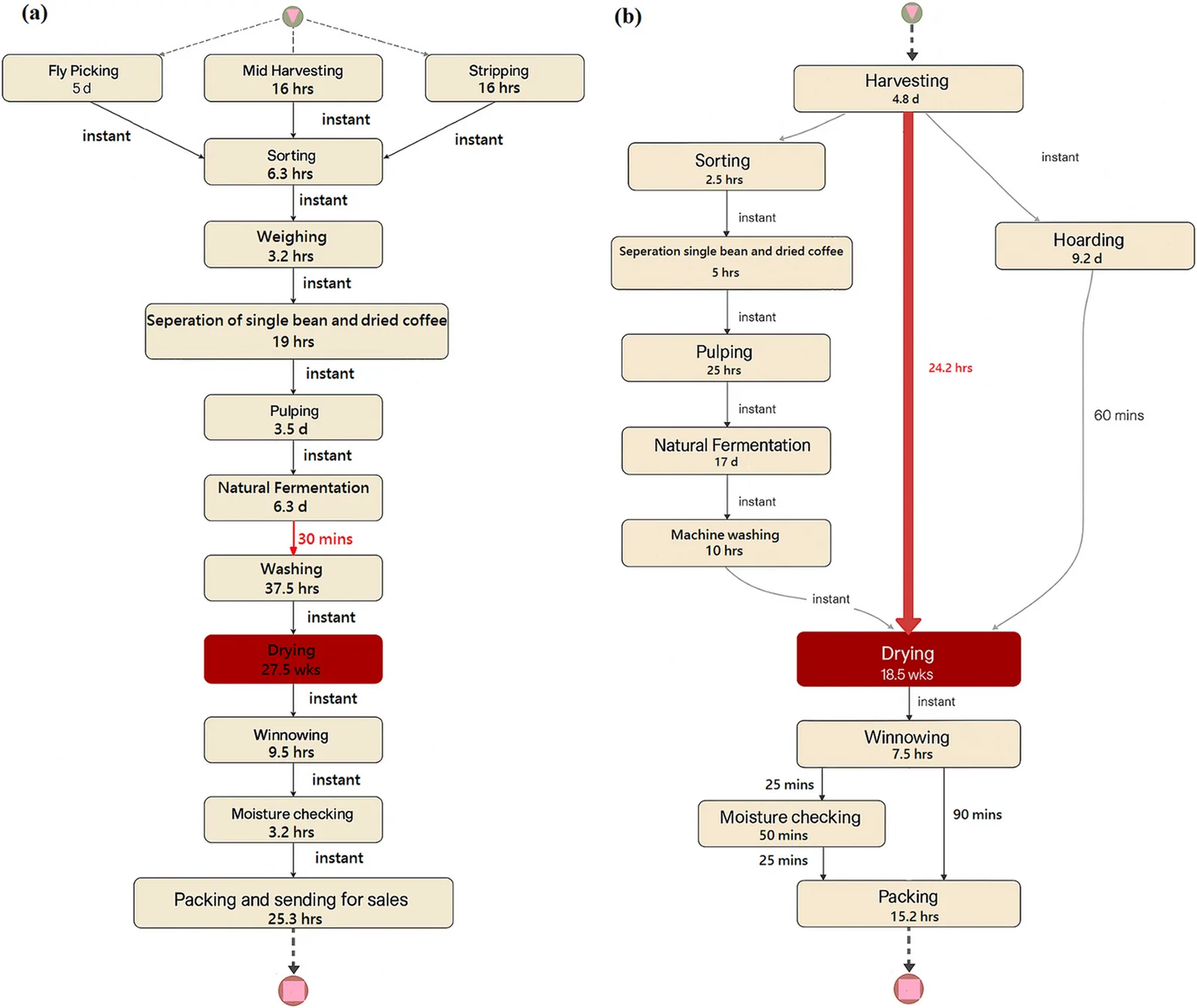
The process model depicting the total duration or time taken for each activity in all 15 cases of (**a**) Arabica harvesting (**b**) Robusta harvesting

Performance metrics such as activity duration were obtained using the Disco tool, where the total time for each activity is displayed within the workflow (Fig. 8). The results show that drying accounts for the largest share of the total process time in both cases, approximately 27.5 weeks for Arabica and 18.6 weeks for Robusta, while harvesting requires about 16 h and 4.8 days, respectively (Fig. 8(a)–(b)). The remaining activities require comparatively less time. Drying is mainly influenced by climatic conditions and involves minimal labour input, whereas harvesting is labour-intensive and represents a major share of human effort. In this study, labour input is quantified in terms of total working hours rather than the number of workers, providing a more accurate representation of workforce contribution across activities.

**Fig. 9:**
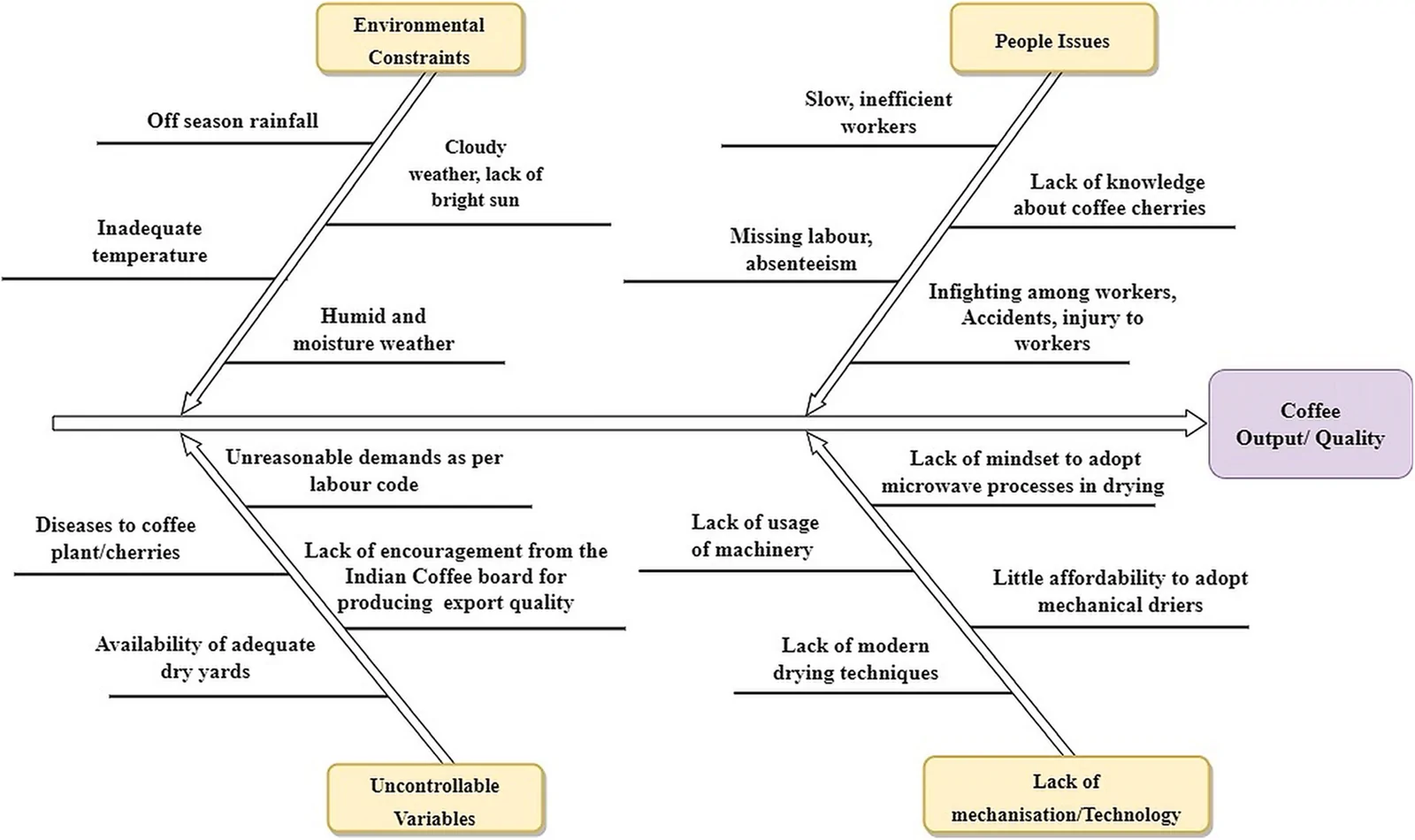
Causative variables related to Arabica and Robusta coffee harvesting

Figure 9 presents the causative variables associated with both Arabica and Robusta coffee harvesting processes. The Ishikawa diagram identifies key factors influencing coffee output and quality, including environmental constraints, people-related issues, uncontrollable variables, and lack of mechanisation/technology. These factors collectively contribute to variations in process efficiency and product quality. Among them, people-related issues were observed to be one of the major contributors, second only to uncontrollable variables, highlighting the role of labour-related aspects such as skill and coordination. The diagram also indicates a clear association between environmental conditions and variations observed in the harvesting process, particularly between Variants 3 and 4 in Fig. 5, where differences in drying duration are evident. Overall, the results show that multiple interconnected factors influence coffee harvesting, with environmental and labour-related variables emerging as key contributors to process variation.

The analysis of workers and the quantity of coffee harvested was carried out using statistical techniques, including Pearson correlation, t-test, ANOVA, and chi-square tests, to examine variations in coffee plucking influenced by different factors. The results show that a correlation test conducted on fifteen batches of fly-picking Arabica coffee reveals a strong negative relationship between the number of workers and the total kilograms plucked (Table 2), indicating that variations in output are associated with workforce size. The statistical analysis was performed at the individual worker level, where each worker’s plucking record was treated as a separate observation, resulting in a total of 504 observations. Although the data collection was organised based on fifteen batches, the shift from batch-level aggregation to worker-level analysis enabled more accurate and statistically reliable inferences regarding productivity patterns.

**Table 2:** The correlation between total kg plucked in fifteen batches of fly picking and number of workers

Particular		Total Kgs plucked	No of workers
Total kg plucked	Pearson Correlation	1	− 0.787^**^
	Sig. (2-tailed)		0.000
	N	504	504
No of workers	Pearson Correlation	− 0.787^**^	1
	Sig. (2-tailed)	0.000	
	N	504	504

*Correlation is significant at the 0.01 level (2-tailed)

The results indicate a statistically significant relationship between total kilograms plucked and the number of workers (*p* < 0.01, α = 0.01), with a Pearson correlation coefficient of − 0.787. This reflects a strong negative linear association, indicating that batches with a larger number of workers tended to record lower total harvesting output. It is important to note that this finding represents an observed association within the dataset and should not be interpreted as a direct causal relationship without further controlled analysis. Multiple confounding factors including field density, worker skill composition, terrain characteristics, and harvesting zone allocation may simultaneously influence both workforce deployment decisions and productivity outcomes.

**Table 3:** Model summary

	R	R Square	Adjusted R Square	Std. Error of the Estimate
1	.787^a^	0.619	0.618	62.431

^a^Predictors: (Constant), No of workers

A regression analysis was performed to find the extent to which the independent variable influences the dependent variable (i.e., the total amount of coffee harvested in fifteen batches of fly harvesting). This statistical method was employed to quantify the correlation between the response and the predictor variables as well as determine the direction and strength of the correlation.

**Table 4:** ANOVA^a^

Model		Sum of Squares	df	Mean Square	F	Sig.
1	Regression	3182163.581	1	3182163.581	816.448	.000^b^
	Residual	1956579.276	502	3897.568		
	Total	5138742.857	503			

^a^Dependent Variable: Total kg plucked in Fifteen batches of fly picking

^b^Predictors: (Constant), No of workers

The results of the comprehensive statistical analysis of the regression analysis are presented in Tables 3 and 4, and 5. The model fit statistics are summarised in Table 3, where the correlation coefficient and the percentage of variation explained by the model is also included. The results of the ANOVA, which test the overall significance of the regression model, are given in Table 4. Interpreting the linear relationship of the independent and dependent variables can be made possible due to Table 5 that describes the regression coefficients computed as well as the intercept and slope values.

**Table 5:** Coefficients ^a^

Model		Unstandardized Coefficients		Standardized Coefficients	t	Sig.
		B	Std. Error	Beta		
1	(Constant)	733.758	17.478		41.982	0.000
	No of workers	−22.558	0.789	− 0.787	−28.574	0.000

^a^Dependent Variable: Total kg plucked in Fifteen batches of fly picking

The regression analysis yielded an R² value of 0.619 (adjusted R² = 0.618), indicating a moderate level of explanatory power, with 61.9% of the variation in fly picking explained by the model. A simple linear regression (y = a + bx) was used to examine the relationship between the number of workers and the total quantity plucked across fifteen batches, resulting in the equation y = 733.758 − 22.558x, which reveals a negative association between workforce size and output. This inverse relationship may be attributed to operational inefficiencies such as worker congestion, variability in worker skill levels, or coordination challenges during harvesting activities, which can reduce individual productivity as workforce size increases. These findings suggest that workforce size alone does not determine output and highlight the influence of additional operational and environmental factors, which were further examined using t-test, chi-square test, and ANOVA to assess their significance.

The results presented in Table 6 indicate that male workers exhibit higher mean plucking values (83.79 ± 5.21 and 59.89 ± 3.33) compared to female workers, while female workers show lower standard deviation, reflecting more consistent performance. Further, the independent samples t-test results in Table 7 (t = 59.218, df = 502, α = 0.05) confirm a statistically significant difference between gender and the kilograms plucked during fly picking. This indicates that the observed variation in output between male and female workers is statistically significant and not due to random variation. Additionally, the validity of this association was further supported using the chi-square test.

**Table 6:** Group statistics

Particular	Gender of the worker	*N*	Mean	Std. Deviation	Std. Error Mean
kg plucked by the worker	Male	285	83.789	5.209	0.309
	Female	219	59.886	3.333	0.225

**Table 7:** Independent samples t-test showing relationship between gender of the workers and the kg plucked

Particular		Levene's Test for Equality of Variances		t-test for Equality of Means						
		F	Sig.	t	df	Sig. (2-tailed)	Mean Difference	Std. Error Difference	95% Confidence Interval of the Difference	
									Lower	Upper
kg plucked by the worker	Equal variances assumed	143.524	.000	59.218	502	.000	23.903	.403	23.110	24.696
	Equal variances not assumed			62.568	487.1	.000	23.903	.382	23.152	24.654

It can be inferred from Table 8 that the quantity of coffee plucked by male workers ranged from 75 kg to 95 kg per batch, with recorded values of 75, 80, 85, and 95 kg. This distribution indicates a comparatively higher plucking capacity among male workers within the observed sample. In contrast, all 219 female workers (100% of the female sample) recorded plucking quantities within a narrower range of 55 to 65 kg, with the majority clustered around 55 kg and 60 kg and a maximum of 65 kg. The data presented in Table 8 thus demonstrate a clear variation in plucking output between male and female workers, both in terms of range and upper limit. While male workers exhibited a broader distribution and higher maximum values, female workers showed a more consistent but comparatively lower output range. These observations provide a quantitative basis for understanding workforce productivity patterns in the fly-picking process.

**Table 8:** Cross tabulation on gender of the worker * kg plucked by the worker

Particular			kg plucked by the worker							Total
			55.00	60.00	65.00	75.00	80.00	85.00	90.00	
Gender	Male	Count	0	0	0	28	113	44	100	285
		% within Gender of the worker	0.0%	0.0%	0.0%	9.8%	39.6%	15.4%	35.1%	100.0%
		% of Total	0.0%	0.0%	0.0%	5.6%	22.4%	8.7%	19.8%	56.5%
	Female	Count	51	122	46	0	0	0	0	219
		% within Gender of the worker	23.3%	55.7%	21.0%	0.0%	0.0%	0.0%	0.0%	100.0%
		% of Total	10.1%	24.2%	9.1%	0.0%	0.0%	0.0%	0.0%	43.5%
Total		Count	51	122	46	28	113	44	100	504
		% within Gender of the worker	10.1%	24.2%	9.1%	5.6%	22.4%	8.7%	19.8%	100.0%
		% of Total	10.1%	24.2%	9.1%	5.6%	22.4%	8.7%	19.8%	100.0%

Table 9 shows that there is a significant correlation between the number of workers and the kg plucked (Pearson’s chi-square (χ^2^) = 504.000, df = 6, α = 0.0.05). Although the survey collected data concerning the age and the number of hours worked per day of the workers, all of the labourers included in the analysis worked harvesting under comparable working hour conditions. Despite slight differences in ages, there was no discernible variance in the amount of time provided for work which would have significant impact on the estimates of total productivity. Fisher’s exact test value is not considered because 0 cells (0.0%) has an expected value that is less than 5. Based on the overall quantity of kilogrammes harvested, worker efficiency was classified as “above average” and “below average.” Male employees were considered “average” if they had a harvest of 80 ± 5 kg, “above average” if they had a harvest of 85 kg and “below average” if they had a harvest of less than 75 kg. Female workers were considered “average” if they pluck 55 ± 5 kg, “above average” if they pluck 60 kg and “below average” if they pluck less than 50 kg. This provides a helpful and reproducible basis for assessing employee performance within the work environment.

**Table 9:** Chi-square test showing the association between the gender and the kg plucked by the worker

Particular	Value	df	Asymp. Sig. (2-sided)	Monte Carlo Sig. (2-sided)			Monte Carlo Sig. (1-sided)		
				Sig.	95% Confidence Interval		Sig.	95% Confidence Interval	
					Upper Bound	Lower Bound		Lower Bound	Upper Bound
Pearson Chi-Square	504.000^a^	6	0.000	.000^b^	0.000	0.006			
Likelihood Ratio	690.025	6	0.000	.000^b^	0.000	0.006			
Fisher’s Exact Test	657.970			.000^b^	0.000	0.006			
Linear-by-Linear Association	440.011^c^	1	0.000	.000^b^	0.000	0.006	.000^b^	0.000	0.006
N of Valid Cases	504								

^a^0 cells (0.0%) have expected count less than 5. The minimum expected count is 12.17

^b^Based on 504 sampled tables with starting seed 2,000,000

^c^The standardized statistic is −20.976

To understand the influence of rain and bad weather (independent variable) one-way ANOVA was applied and the results are depicted in Table 10. The result of ANOVA from Table 10 shows F = 40159.766, df = 2, *p* < 0.05 and is significant.

**Table 10:** ANOVA test showing the association between the weather and total kg plucked

Particular		Sum of Squares	df	Mean Square	F	Sig.
Total kg plucked without rain	Between Groups	123759625.976	2	61879812.988	40159.766	0.000
	Within Groups	771961.326	501	1540.841		
	Total	124531587.302	503			
Total kg plucked with rain	Between Groups	1754104.808	2	877052.404	187.243	0.000
	Within Groups	2346694.745	501	4684.021		
	Total	4100799.554	503			

Rainy, non-rainy and bright, sunny weather have statistically significant effects on the total number of kilogrammes that the workers harvest, according to the results of the post hoc multiple comparison test (Table 11). The investigation validates the relevance of environmental factors in driving field production by showing that changes in climatic conditions are associated with quantifiable difference in labour output. The level of efficiency of workers, which was used to separate them into above-average and below-average ones, was one of the intervening variables that were considered in addition to the main independent variables. This categorisation made it possible to assess the differences in productivity of the workforce more precisely. A paired t-test was used to further look into whether weather has a significant effect on above-average workers work efficiency, the results can be seen in Table 11.

**Table 11:** Post hoc multiple comparisons

LSD							
Dependent Variable	(I) Rain and wet weather	(J) Rain and wet weather	Mean Difference (I-J)	Std. Error	Sig.	95% Confidence Interval	
						Lower Bound	Upper Bound
Total kg plucked without rain	Rain	No rain	−500.000^*^	4.411	0.000	−508.667	−491.333
		Bright	−1161.878^*^	4.109	0.000	−1169.952	−1153.805
	No rain	Rain	500.000^*^	4.411	0.000	491.333	508.667
		Bright	−661.878^*^	4.427	0.000	−670.576	−653.181
	Bright	Rain	1161.878^*^	4.109	0.000	1153.805	1169.952
		No rain	661.878^*^	4.427	0.000	653.181	670.576
Total kg plucked with rain	Rain	No rain	147.107^*^	7.691	0.000	131.996	162.218
		Bright	82.003^*^	7.165	0.000	67.926	96.080
	No rain	Rain	−147.107^*^	7.691	0.000	−162.218	−131.996
		Bright	−65.104^*^	7.719	0.000	−80.269	−49.940
	Bright	Rain	−82.003^*^	7.165	0.000	−96.080	−67.926
		No rain	65.104^*^	7.719	0.000	49.940	80.270

*The mean difference is significant at the 0.05 level

Table 12 indicates that the mean quantity of coffee plucked by above-average workers under non-rainy conditions is comparatively higher, with a recorded mean of 33.5714 ± 9.53923 kg, whereas under rainy conditions the mean output decreases to 18.5714 ± 6.93214 kg. This noticeable difference in mean values suggests that the absence of rainfall contributes to improved work efficiency and higher productivity levels among above-average workers. The relatively larger standard deviation observed in non-rainy conditions also reflects variability in individual performance, although the overall output remains substantially greater than that recorded during rainy conditions. These findings highlight the influence of weather conditions on worker productivity, particularly among those categorized as above-average performers.

**Table 12:** Paired samples statistics

Particular		Mean	*N*	Std. Deviation	Std. Error Mean
Pair 1	kg Plucked by Above Average worker without rain	33.571	504	9.539	0.4249
	Above average worker after rain	18.571	504	6.932	0.3088

The paired sample correlation analysis (Table 13) indicates a strong positive relationship between the variables (*r* = 0.889). The *p* < 0.05 confirms that the correlation is statistically significant at α = 0.05. The results show that in the absence of rainfall, above-average workers consistently achieve higher harvesting output (kg), indicating that favorable weather conditions are associated with increased and predictable productivity levels.

**Table 13:** Paired samples correlations

Particular		*N*	Correlation	Sig.
Pair 1	kg Plucked by Above Average worker without rain & Above average worker after rain	504	0.889	0.000

The paired sample t-test results (Table 14) show a t-value of 72.674 with 503 degrees of freedom (df). Since *p* < 0.05 at α = 0.05, the difference is statistically significant. The results indicate a significant variation in the total kilograms plucked by workers during fly picking under different weather conditions, confirming that rainfall has a measurable effect on worker productivity.

**Table 14:** Paired samples test showing the kg plucked by above average worker

Particular		Paired Differences					t	df	Sig. (2-tailed)
		Mean	Std. Deviation	Std. Error Mean	95% Confidence Interval of the Difference				
					Lower	Upper			
Pair 1	kg Plucked by above average worker without rain - above average worker after rain	15.000	4.634	.206	14.594	15.406	72.674	503	.000

The PM analysis revealed that drying and harvesting are the most time-intensive activities in both Arabica and Robusta coffee harvesting workflows. Drying duration varied across batches, indicating differences in processing time. Statistical analysis using regression and Pearson correlation showed a significant negative linear relationship between the number of workers and the quantity of coffee harvested during the fly-picking process. In addition, gender-based differences were observed, with male workers showing higher average output compared to female workers.

## Discussion

Integrating PM with statistical analysis, the present paper provides a comprehensive and methodologically well-grounded examination of coffee harvesting processes, both operational and analytical understanding of the dynamics of the workflow and productivity aspects. The results indicate that the process duration varies to a great extent, particularly in the harvesting and drying stage, although the sequence of activities in Arabica harvesting systems and Robusta harvesting systems can be the same. As in previous process-based studies, this helps to sustain the idea that diversity in agricultural processes is primarily due to contextual and external factors, but not process differences [39, 40]. The most time-consuming part is drying, which adds about 7 days and 8 h to an overall time of 8 days and 10 h in a typical Arabica sample, based on the empirical evidence of PM analysis. This evidently makes drying one of the bottlenecks. Such a process time concentration is indicative of a structurally sensitive stage where process efficiency is rigidly controlled by environmental variability instead of merely being descriptive. The sensitivity of the system to environmental factors such as solar radiation and humidity, which influence the rate of moisture removal and by extension the general throughput, is shown in the difference in time taken to dry the less-dried process variant. These findings are consistent with the past studies that indicate that drying is a climate-sensitive process that influences the quality and efficiency of products [41, 42].

The identification and interpretation of the inverse relationship between workforce size and overall productivity constitute an important contribution of this study, supported by both theoretical reasoning and empirical evidence. Statistical analysis revealed a strong negative correlation between the number of workers and output (*r* = −0.787), indicating that larger workforce size does not necessarily lead to increased productivity. This relationship was further validated through regression analysis, where the model demonstrated good explanatory power (R² = 0.619) along with a highly significant F-value (816.448), confirming the statistical reliability of the relationship. The findings suggest that factors such as labour efficiency, coordination among workers, skill variation, and field operating conditions may play a significant role in determining productivity, highlighting the need to optimize workforce management rather than simply increasing labour numbers. According to the regression equation (y = 733.758 − 22.558x), it can be deduced that, in this sample data set, an increment in the number of workers leads to a decrease of about 22.56 kg in the total production. Although there is strong statistical evidence of this correlation (R² = 0.619, F = 816.448, *p* < 0.001), it must be understood properly. This might be partially due to the effect of confounding variables like division into sections, differences in planting density, or the propensity of estate managers to assign more workers to areas where yields are less. Instead of claiming causation, these results show that, by itself, labor force is not an effective determinant of productivity and many other factors contribute to determine harvests. An analytical explanation of this relationship can be found with the theory of diminishing marginal productivity, which postulates that expansion of labour beyond an optimal degree will result in reduced efficiency because of congestion, overlapping of harvesting zones and coordination inefficiencies. The disproportional geographical placement of ripe cherries in fly picking imposes a constraint of binding labour usage leading to competition between workers and reduction in individual efforts. The size of this relationship and its statistical significance justify the notion that it is a systemic phenomenon and not an oddity of the situation. This description is also in line with the established studies in the field of agricultural economics that indicate that high labour concentration leads to inefficiencies and reduced marginal returns [43, 44].

While significant differences existed between the productivities of men and women, such results need to be analyzed with caution and in context. To attribute any differences to capability differences due to biological differences between the sexes is both inappropriate and scientifically incorrect. The difference can more likely be attributed to a combination of structural and procedural issues particular to the plantation system harvesting setting. The results indicated that male workers recorded a higher average productivity (83.79 ± 5.21 kg) compared with female workers (59.89 ± 3.33 kg), with the difference being statistically significant (t = 59.218). However, the distributional characteristics of the data suggest important distinctions in performance variability, as female workers exhibited a comparatively narrower productivity range (55–65 kg), indicating greater consistency in output, whereas male workers demonstrated a broader productivity range (75–95 kg), reflecting higher variability in performance levels. These observations indicate that the recorded productivity differences are influenced by several interconnected operational and workplace factors rather than gender alone. In plantation-based harvesting systems, differences in task allocation and physical workload distribution may substantially affect worker output, as labourers are often assigned varying picking areas, terrain conditions, crop densities, and levels of physically demanding activities. In addition, factors such as field conditions, work experience, ergonomic demands, and access to operational resources can further influence productivity outcomes [45, 46].

The relatively consistent productivity observed among female workers further highlights the importance of reliability and process stability in labour-intensive plantation operations. Consistency in harvesting performance contributes to predictable workflow management and operational efficiency, both of which are critical for effective plantation management. The present findings are consistent with earlier studies reporting that gender-related variations in agricultural productivity are primarily shaped by institutional, socioeconomic, and occupational factors rather than biological determinants [47–49]. Therefore, the observed productivity patterns should be interpreted as reflecting the influence of workplace structure and operational conditions within coffee plantation systems.

There is solid statistical evidence that environmental conditions are one of the key factors that influence the productivity and process efficiency. In accordance with the ANOVA results (F = 40159.766), the weather has very great influence on overall output. Post hoc comparisons find statistically significant differences in the level of productivity with variations in climatic circumstances. The paired sample test results indicate that there are always weather differences that influence performance of the workers since the correlation between the two is strong (*r* = 0.889) and the t-test value is very significant (t = 72.674). In line with other researches cited in the literature [50, 51], these findings indicate that the productivity is dependent on worker efficiency and environmental constraints together as opposed to labour factors. This confirms the hypothesis that agricultural systems are coupled human-environment interactions with field activities and post-harvest operations directly influenced by climate variability.

The process perspective of PM technologies provides invaluable information about the dynamics of operations and variability of workflows. Although the sequences of activity are similar, the identification of multiple variations of processes prompts the idea that the process execution is dynamic in nature and is affected by the context. The contrast between the less rigid workflows of Robusta and the managed workflow of the Arabica makes the impact of processing methods and environmental conditions on consistency and efficiency even more apparent. In line with results in other fields, the ability of PM techniques to visualize these changes and measure durations of activities provides a significant methodological supplement that can be extrapolated to complex agriculture systems [52, 53].

Operational wise, the findings are conclusive that the more emphasis should be on the optimisation of the present resources than adding more labour. The statistically significant negative relationship between the workforce size and production indicates the need to efficiently coordinate and allocate labour. Also, the great role of environmental conditions underlines the importance of climate-sensitive operation models, including the implementation of more efficient drying methods to reduce the variability of the processes, and the alignment of harvesting periods with weather conditions. These approaches are in accordance with the broader bioeconomy paradigm, which focuses more on the use of data and sustainable use of resources [54].

One should be aware of drawbacks of this research. The findings of this study should be interpreted considering certain limitations related to measurement and sampling. The analysis was conducted using data collected from a limited number of coffee estates within a specific region of Karnataka during a single harvesting season, which may restrict the generalizability of the results to other geographical locations, plantation conditions, or harvesting periods. In addition, although appropriate validation and cross-verification procedures were adopted during data collection, the reliance on manual plantation records and field observations may introduce minor errors, inconsistencies, and recall bias in the recorded information. These factors could influence the precision of the measured variables and should be considered while interpreting the results. Nevertheless, as the study was confined to a specific geographical region and harvesting season, the findings should primarily be interpreted within plantation environments exhibiting comparable operational and agro-climatic conditions. Differences in plantation management practices, terrain characteristics, labour organisation, climatic variability, and harvesting schedules across regions may affect the direct transferability of the observed relationships.

Future studies may further strengthen the robustness and wider applicability of the findings by incorporating larger and more heterogeneous datasets spanning multiple geographical regions and harvesting periods. The integration of automated and sensor-based data acquisition systems, along with digital tracking technologies, could improve data accuracy and facilitate real-time operational analysis. In addition, the application of PM techniques in combination with advanced analytical and operational management tools may provide deeper insights into workflow inefficiencies and support the development of scalable strategies for improving labour productivity and optimising coffee harvesting operations across diverse plantation systems.

## Conclusion

This paper demonstrates how Process Mining (PM) can be utilized as a data-driven approach to assessing coffee harvesting activities by systematically mapping workflows in Arabica and Robusta processing in the field. It is found that the processing of Robusta is more varied due to the presence of operational and other environmental factors whereas Arabica harvesting is relatively organised. The labour-intensive tasks, particularly, the plucking, were observed to have a significant effect on harvesting efficiency, which explains the importance of efficient staff deployment and planning.

In practice, the findings suggest that productivity at the estate level can be improved by improving workforce management, coordination of activities and selective application of mechanisation. Although adaptive solutions are needed to deal with variability in Robusta processing, operations, particularly wet processing, can be standardised to better deal with operational consistency. Another focus of the paper is the role of the solutions based on data in monitoring and making informed decisions in plantation systems to give the relevant information to practitioners and policymakers. The research provides the foundation of future investigations based on larger data sets and more advanced analysis perspectives, but is limited to a limited number of Karnataka estates and focuses primarily on the discovery aspect of PM. Overall, the study advances the use of PM in agriculture and offers useful suggestions for raising coffee harvesting systems’ productivity.

## Data Availability

The original contributions presented in the study are included in the article. Further inquiries can be directed to the corresponding author.
